# Risk of Migraine in Europeans with Low Melanin Levels—A Population Based Case-Control Study

**DOI:** 10.3390/brainsci12050620

**Published:** 2022-05-10

**Authors:** Magdalena Kobus, Elżbieta Żądzińska, Aneta Sitek, Jacek Pełka, Jacek J. Rożniecki, Bogusław Antoszewski

**Affiliations:** 1 Department of Anthropology, Faculty of Biology and Environmental Protection, University of Lodz, 90-237 Lodz, Poland; elzbieta.zadzinska@biol.uni.lodz.pl (E.Ż.); aneta.sitek@biol.uni.lodz.pl (A.S.); 2 Biological Anthropology and Comparative Anatomy Research Unit, School of Medicine, University of Adelaide, Adelaide 5005, SA, Australia; 3 Department of Neurology, Norbert Barlicki Memory University Teaching Hospital, 90-153 Lodz, Poland; jacekpelka@neurocenter.pl; 4 Department of Neurology, Stroke and Neurorehabilitation, Medical University of Lodz, 90-153 Lodz, Poland; jacek.rozniecki@umed.lodz.pl; 5 Department of Plastic, Reconstructive and Aesthetic Surgery, Institute of Surgery, Medical University of Lodz, 90-153 Lodz, Poland; boguslaw.antoszewski@umed.lodz.pl

**Keywords:** migraine, melanin, headache, skin pigmentation, cutaneous colorimetry

## Abstract

Populations with a relatively low concentration of melanin, e.g., inhabitants of Europe, North America, and Australia, are the most vulnerable to the harmful effects of UV radiation. Individuals with fair phototype are at greatest risk of developing skin cancer. Several neurological studies present that light skin may modify the risk of Alzheimer’s and Parkinson’s diseases. However, the relationship between migraine and skin pigmentation has not been investigated yet. The objective of this study is to provide evidence of the relationship between skin pigmentation and migraine prevalence in adults. We examined a group of 148 adults (33 men, 115 women) with migraine and a control group of 107 adults (43 men, 64 women). Parameters of skin pigmentation (melanin index, erythema index, CIElab, and RGB scales) were measured using a DSM II Cortex Technology dermospectrophotometer. Risk of migraine in lightly pigmented adults was elevated. Individuals with a low melanin index had over 3-fold increased risk of migraine (women: OR 3.53, men: OR 3.73). Fair phototype, which results from lightly pigmented skin, was associated with migraine prevalence. Migraineurs should take extra care to protect their skin from the negative effects of solar radiation.

## 1. Introduction

Melanin is a heterogeneous group of natural pigments. Melanins differ in terms of origin, color, composition, and functions they perform in organisms [1,2,3]. In humans, they are mainly known to be responsible for pigmentation of the skin, hair, and iris [4,5,6], although one type of melanin (neuromelanin) occurs in the central nervous system and does not affect pigmentation phenotype [7,8,9]. Melanins in animals are assigned a wide variety of biological functions. In invertebrates, pheomelanin production has not been found, while eumelanin can protect against harmful solar radiation and against predators [10]. In humans, melanin is found in many different organs: the skin, iris, hair, and some areas of the brain. The presence in the skin and the iris can be closely related to the photoprotective role, while the presence in brain areas is still not fully understood. Neuromelanin is known to have the ability to bind various potentially harmful substances, such as ions of certain metals, drugs, and pesticides [11]. It has been shown that under certain conditions, neuromelanin may have a neurodegenerative effect [12,13]. Its role in the etiology of Parkinson’s disease (PD) is also suggested [13]. Identifying pre-motor symptoms is crucial for early diagnosis and initiating neuroprotective treatment, because another possible therapy in PD is symptomatic. Jucevicute et al. reported certain skin and hair features (e.g., earlier age at onset of hair greying) might be considered risk factors of PD [14]. 

Phototype depends on the quantity of melanin produced by melanocytes. The number of epidermal melanin units is similar in humans irrespective of ethnicity [15,16,17]. Differences in skin pigmentation depend on pigment levels, are regulated by genetic and endocrine factors and can be induced by UV radiation [6,18,19,20,21,22,23,24].

Skin diseases and conditions might result in disturbances of human pigmentation (hyperpigmentation, hypopigmentation, or depigmentation) [17]. It is widely known that fair skin is linked to an increased risk of skin cancer [25,26]. Furthermore, light skin is related to an increased risk of neurological diseases like Alzheimer’s (AD) and PD, the most frequent neurodegenerative diseases [14,27,28,29]. Population-based studies indicated that fair skin is responsible for simultaneous increased risks of cutaneous melanoma and PD [30]. The mechanism of these relations is connected to the disorders of melanogenesis pathway. One of the basic genes involved in melanogenesis is the melanocortin-1 receptor (MC1R). MC1R is involved in the pathogenesis of PD and is responsible for the co-occurrence of PD and cutaneous melanoma [31]. The loss of the melanocortin-1 receptor function is connected to an elevated basal pain threshold, as well as greater sensitivity to opioid analgesics [32]. Furthermore, clinical research shows a correlation between fair skin and abnormally enlarged echogenicity of the substantia nigra [27]. 

Migraine is a common neurological disease identified as the world’s second leading cause of disability that affects a large percentage of the global population [33,34]. It involves prenatal, genetic, and environmental factors [35,36,37]. The main feature of the condition is moderate to severe headache, which is felt as a throbbing pain on one side of the head [38,39]. Diagnosis is based on symptoms that may include nausea, vomiting, light/sound sensitivity, and often visual, sensory, or speech symptoms [40]. Each new contribution to migraine research seems to be highly desirable, because migraine affects over 1 billion people worldwide [41].

Limited knowledge of skin-pigment-related and neurological diseases makes migraine an interesting field of study of their relationship. So far, it has been demonstrated that migraine has been associated with alopecia areata [42], atopic dermatitis [43], psoriasis [44], and rosacea [45,46,47]. Temporary skin changes during migraine attacks have been reported in a few case studies (the oldest one speculated to be from 1961), but the causes of these events remain unknown [48,49,50]. Putative association of skin pigmentation and migraine has not been investigated yet. 

The Fitzpatrick skin phototype (FSP) classification scale is the traditional way of human skin color classification, divided into six categories. To avoid subjective skin evaluation, we used an objective quantification method which can predict skin cancer better than traditional skin phototyping [51]. Cutaneous colorimetry is a quick and simple non-invasive technique for skin color assessment, using sensitive and reliable modern instruments such as a dermospectrophotometer. Color systems (such as CIELab) help to make a comprehensive skin research tool using colorimetric devices [52,53]. 

Our study offers a new perspective to investigate whether skin pigmentation pattern correlates with the risk of migraine in adults.

## 2. Materials and Methods

The study was conducted according to the guidelines of the Declaration of Helsinki and approved by the Ethical Commission at the University of Lodz (no. 16/KBBN-UŁ/III/2018). Data collection started in 2019 and ended in 2020. Written consent forms were obtained from participants after they received detailed study information. Participants were recruited at Norbert Barlicki Memory University Teaching Hospital No. 1 in Lodz (Neurological Clinic and Plastic Surgery Clinic). The study included two groups of adults: individuals with migraine (148 participants—115 women and 33 men, aged 19–76 years), and a control group (107 participants—64 women and 43 men aged 21–74 years). In both study groups, participants denied history of skin diseases, skin-related autoimmune disorders, and skin cancer. Participants declaring sunbathing or using artificial sources of UV exposure (e.g., sunbeds) within the last 3 months were excluded from the study (3 women from the study group and 2 women and 2 men from the control group). Participants were examined by a neurologist and categorized into migraine group (according to the International Classification of Headache Disorders, 3rd edition) or neurological-diseases-free control group [40]. 

FSP classification was created in 1975 [54]. Although FSP scale is a standard method of assessing skin color widely used in clinical studies, recent data suggest that self-reported phenotype information is inaccurate [55]. The FSP skin assessment method was based on patient-reported responses to questions regarding skin reaction to sun exposure (skin erythema and tanning reactions). Numerical values were assigned to each answer which in total corresponds to the one of the six Fitzpatrick skin phototypes. The author of FSP noted in 1988 that standardizing skin assessment is a challenging task and in the future, a more objective method should be created that is simple as well as practical [54]. In this study, we used a simple, rapid, and non-invasive method. The dermospectrophotometer is the device which provides colorimetric data in a more accurate and reliable manner. 

Parameters of skin pigmentation (MI—melanin index, EI—erythema index, L, *a*, *b*, R, G, B) were measured at the inner part of both arms using a DSM II Cortex Technology dermospectrophotometer, 6 times in total (3× left arm, 3× right arm). Buttocks and arms are the most often used sun-unexposed anatomical sites and present skin color equally [56]. We found the inner arm measurement more comfortable for participants; therefore, skin assessment was easy to conduct in both children and adults. The dermospectrophotometer was calibrated before every use. An average value obtained on the basis of six measurements was used for the analyses. Skin color can be quantified through color organization systems, e.g., CIElab color space. The CIElab shows colors’ lightness (L*), red/green intensity (a*), and yellow/blue intensity (b*) values, respectively [53]. Next to the CIElab, we used additive color model RGB, which refers to primary colors (R—red, G—green, B—blue) [57].

Statistical analysis was performed in the STATISTICA 13.0 program. All calculations were carried out in spreadsheets after anonymization of the participants’ data. Analysis of covariance (ANCOVA) was used to assess differences in the pigmentation parameters in both study groups (age was included as a covariate). At the ROC curve and Youden index analysis, the cut-off points for melanin indexes were defined. Odds ratios were calculated using the logistic regression model. 

## 3. Results

Mean age for women (F) was 42.6 years in the migraine group and 43.7 years in the control group (Table 1). Mean age for men (M) was 41.5 years in the migraine group and 41.1 years in the control group (Table 2). No statistically significant demographic differences were found between the migraine and control groups in men and women. 

Statistical analysis showed that most of the spectrophotometric skin color parameters differed significantly between study groups. Mean values of MI in the migraine group were 27.08 for women and 27.07 for men. Mean values of MI in the control group were 28.57 for women and 28.27 for men (Table 1 and Table 2). ANCOVA analysis showed significant relationships between lower MI and prevalence of migraine in women and men (*p* = 0.002, *p* = 0.032) (Table 3 and Table 4). Significantly lower MI results in fairer skin color.

Cut-off criteria for melanin level were <28.56 MI in women and <27.68 MI in men. Women with MI < 28.56 had 3.5-fold increased risk of migraine (OR 3.53, 95% CI 1.80–6.90, *p* < 0.001). Men with MI < 27.68 had 3.7-fold increased risk of migraine (OR 3.73, 95% CI 1.43–9.73, *p* = 0.007). 

Comparison of spectrophotometric skin color parameters in both study groups showed that women with migraine also had a lower erythema index (EI, *p* < 0.001), higher values of L* (*p* = 0.002), lower values of a* (*p* = 0.001), and higher values of R/G/B (R, *p* = 0.002; G, *p* = 0.002; B, *p* = 0.004) (Table 3). Lower erythema index (skin redness), higher parameter of skin lightness (L*), lower red/green intensity (a*), and higher values of R/G/B parameters (effect of less color saturation) clearly indicated fair phototype pattern in female migraineurs.

Among all parameters, only two (EI, *p* = 0.008; a*, *p* = 0.007) were age-related, and this association was observed only in men (Table 4). Comparison of spectrophotometric skin color parameters in both study groups showed that men with migraine had significantly higher values of R/G (R, *p* = 0.029; G, *p* = 0.043), despite lower MI. A higher value of lightness parameter (L*) was on the border of significance in this group (*p* = 0.052). One limitation of the male study group was its small sample size.

## 4. Discussion

The present study revealed that fair skin is related to migraine—A neurological disorder with severe and disabling symptoms [58,59]. To the best of our knowledge, this is the first study that considered risk of migraine and skin pigmentation. The risk of migraine was 3.5 times increased in light-skinned women (OR 3.53) and 3.7 times increased in light-skinned men (OR 3.73), while melanin index was below cut-off point (F: 28.56, M: 27.68). It is taken that men are more susceptible to skin cancer [60,61,62]. Recently, Antoszewski et al. (2021) published a report that confirms this relationship in Polish population. Women visited the physician sooner than men, and as a result, the size of the tumors was smaller in the women group. This may be due to putative better awareness in women, cosmetic concerns, or both factors [63]. 

Depending on the pheo/eumelanin ratio, skin and hair color is either less or more intensified. The dominant production of pheomelanin is related to the RHC phenotype, which consists of red hair and light irises and skin. Light skin and red hair are regarded as the most important phenotype risk factors of skin cancer [64,65]. The red hair phenotype is also associated with increased pain thresholds (and elevated sensitivity to opioid analgesics) [32]. Meta-analyses revealed lower thermal sensitivity in lightly pigmented skin individuals [66].

Adaptation to the thermal pain is observed in lightly pigmented populations living in Europe and is characterized by mutation of the TRPM8 gene, which is called the sensor of cold temperature. Detecting temperature is a primary task of the nervous system. Variants of TRPM8 have been associated with migraines (GWAS) [67] and its expression is being found (e.g., in trigeminal ganglion) [68]. A variant that increases TRPM8 expression is much more common in Northern Europeans (Finland: 88 %) than in Africans (Nigeria: 5%) [69]. According to the WHO, migraine is rare in Africa and is most common in Europe [70]. TRMP8 plays complex role in pain perception and potentially contributes to the changes in the prevalence of migraine in particular human groups.

Knowledge of melanin and skin pigmentation has been widely analyzed in the evolutionary ecology of humans and animals and in medicine, but analyzing skin pigmentation patterns in headache disorders has been overlooked [71,72]. We paid attention to pigmentation in migraine patients as a little-known field of study with significant practical implications and great potential for further research.

## 5. Conclusions

Our present study shows the problem from a public health perspective, because less immanent protection to UV radiation suggests that migraineurs should pay more attention to using sun-blocking products. In addition to this, bright light is one of many triggers—as well as aggravating factors—of migraine attacks, so individuals with migraine are more susceptible and vulnerable to sunlight in two different ways [73,74]. This presents the problem of migraine as multifactorial, both from a researcher and a patient perspective. 

## Figures and Tables

**Table 1:** Statistical characteristics ($\overset{-}{x}$ ± SD) of age and spectrophotometric skin color parameters in females.

Variable	FEMALES		Student *t*-Test *	
	Migraine Group N = 115	Control Group N = 64	t	*p*
Age	42.55 ± 14.18	43.73 ± 14.65	−0.53	0.597
MI	27.08 ± 2.81	28.57 ± 3.25	−3.21	0.002
EI	9.26 ± 1.65	10.27 ± 1.90	−3.73	0.000
L*	45.99 ± 3.96	43.99 ± 4.11	3.21	0.002
a*	13.61 ± 1.75	14.61 ± 1.96	−3.52	0.001
b*	16.12 ± 3.04	16.17 ± 3.44	−0.11	0.913
R	137.01 ± 8.60	132.58 ± 9.70	3.15	0.002
G	111.07 ± 11.83	105.24 ± 11.23	3.22	0.002
B	106.27 ± 12.34	100.27 ± 13.90	2.98	0.003

* carried out for equal or different variances (depending on the results of the Fischer–Snedecor test).

**Table 2:** Statistical characteristics ($\overset{-}{x}$ ± SD) of age and spectrophotometric skin color parameters in males.

Variable	MALES		Student *t*-Test *	
	Migraine Group N = 33	Control Group N = 43	t	*p*
Age	41.52 ± 15.72	41.07 ± 13.70	0.13	0.896
MI	27.07 ± 2.30	28.27 ± 2.38	−2.21	0.030
EI	9.66 ± 1.63	10.45 ± 2.10	−1.78	0.080
L*	45.55 ± 3.31	44.00 ± 3.45	1.98	0.051
a*	14.72 ± 2.52	15.14 ± 2.80	−0.68	0.500
b*	14.78 ± 2.68	16.74 ± 5.29	−2.10	0.039
R	137.08 ± 7.33	133.26 ± 7.32	2.25	0.028
G	109.88 ± 8.78	105.45 ± 9.62	2.06	0.043
B	104.89 ± 12.06	100.21 ± 12.96	1.61	0.112

* carried out for equal or different variances (depending on the results of the Fischer–Snedecor test).

**Table 3:** ANCOVA results showing differentiation of the spectrophotometric skin color parameters depending on the study group in women (adjusted for age).

Dependent Variable	Independent Variable		Covariate		partial η^2^_Group_
	Study Group		Age		
	F	*p*	F	*p*	
MI	10.01	0.002	1.5	0.222	0.05
EI	13.72	0.000	0.08	0.778	0.07
L*	9.96	0.002	1.97	0.162	0.05
a*	12.49	0.001	0.41	0.525	0.07
b*	0.31	0.581	0.01	0.931	0.00
R	9.63	0.002	1.84	0.177	0.05
G	10.15	0.002	0.24	0.622	0.05
B	8.62	0.004	0.95	0.331	0.05

**Table 4:** ANCOVA results showing differentiation of the spectrophotometric skin color parameters depending on the study group in men (adjusted for age).

Dependent Variable	Independent Variable		Covariate		partial η^2^_Group_
	Study Group		Age		
	F	*p*	F	*p*	
MI	4.80	0.032	0.34	0.561	0.06
EI	3.28	0.074	7.56	0.008	0.04
L*	3.89	0.052	2.16	0.146	0.05
a*	0.44	0.508	7.59	0.007	0.01
b*	3.72	0.058	1.17	0.283	0.05
R	4.97	0.029	0.18	0.672	0.06
G	4.24	0.043	2.36	0.129	0.05
B	2.59	0.112	3.87	0.053	0.03

## Data Availability

The datasets used in the current study are available from the corresponding author on request.
